# The Twenty-Fifth Annual Meeting of the Mississippi Valley Dental Association

**Published:** 1869-03

**Authors:** 


### Proceedings of Societies.

---

### THE TWENTY-FIFTH ANNUAL MEETING OF THE MISSISSIPPI VALLEY DENTAL ASSOCIATION.

WEDNESDAY, MARCH 3RD, 1869.

Met in the Lecture-room of the Ohio Dental College, at 10 A. M.

President Cushing in the Chair.

Members present: Drs. A. Berry, J. Taft, H. McCollum, G. W. Keely, Jas. Taylor, W. H. Shadoan, J. A. McClelland, J. Cheesbrough, H. A. Beamer, A. A. Blount, G. H. Cushing, W. H. Sedgwick, R. A. Mollyneaux, J. G. Cameron, R. L. Evans, W. H. Morgan, W. F. Morrill, W. G. Redman, A. M. Moore, J. A. Watling, Sam'l Wardle, Will Taft, H. R. Smith, H. A. Smith.

Minutes of last session were read and approved.

Intermission was allowed for members to settle their annual dues.

Dr. Taylor offered the following resolution:

*Resolved*, That the Treasurer be authorized to pay Dr. J. Taft thirty-five dollars for reporting the discussions.

Drs. Keely, Moore and Evans, were appointed Committee on Membership, to fill vacancies occurring from absence of members of the regular committee.

The Executive Committee reported the following order of business:

I. Report of Officers.
II. Election of Officers, 3 P. M., second day.
III. Miscellaneous business.
IV. Essays or papers to be read on the opening of discussion.

SUBJECTS FOR DISCUSSION.

1. Use of the file in Operative Dentistry.
2. Treatment and preservation of the deciduous teeth.
3. Mechanical Dentistry.
4. Operative Dentistry.
5. Salivary Calculus. Exhibition of instruments and appliances for one hour, commencing at 1½ P. M., 2d day.

W. H. MORGAN,<br>H. A. SMITH,<br>A. A. BLOUNT, } *Committee.*

The Committee on Membership presented the names of Dr. E. W. Ruth, of Maysville, Ky., and W. R. Johnson, Columbus, Tenn, both were elected.

The Committee to Revise Constitution and By-Laws were discharged, and a new one consisting of Drs. J. Taft, and G. W. Keely, appointed.

The Committee to prepare a popular essay for gratuitous distribution was discharged.

On motion of Dr. H. A. Smith, the sum of fifty dollars ($50) was offered for a popular essay.

A Committee of five, consisting of Drs. H. A. Smith, Jas. Taylor, W. H. Morgan, A. M. Moore, and J. Taft, were appointed to examine into the merits of the essays that may be presented for the above prize.

The Treasurer made the following report upon the condition of the finances of the Association.

*Dr.*

| | |
|---|---|
| Received for Dues and new Members | $ 48 00 |
| " from former Treasurer | 100 00 |
| " Balance due | 139 00 |

*Cr.*

| | |
|---|---|
| Paid Janitor | $ 15 00 |
| " Dr. G. W. Field | 5 95 |
| " Dr. J. Taft, Reporting | 35 00 |
| " for Printing | 5 00 |
| " Appropriation to College | 50 00 |
| | $176 40 |

The Committee, to whom was referred the report of Treasurer, find the same correct.

H. R. SMITH, R. A. MOLLYNEAUX, } *Committee.*

Dr. J. G. Cameron, of the Committee upon revision of the membership roll, reported the list of delinquents, and upon motion of Dr. A. Berry, the Treasurer was instructed to notify delinquents of the amount of dues standing against them.

The Membership Committee presented the name of Dr. E. C. Sloan, of Ironton, Ohio, as a member of the Association, who was elected.

On motion of Dr. Jas. Taylor, It was decided to attend the funeral of Dr. N. Allen, in a body, this afternoon at 2 o'clock.

Adjourned till 2 o'clock, P. M.

### AFTERNOON SESSION.

President and Secretary absent.

Dr. Jas. Taylor was appointed President, and Dr. H. A. Smith, Secretary, *pro tem.*

First subject for discussion, "The use of the file in Operative Dentistry," was taken up and disposed of after considerable discussion.

The second subject, "Management and Preservation of deciduous teeth," was taken up, and after some discussion was laid over till second day.

Adjourned till second day, 10 A. M.

### MORNING SESSION, SECOND DAY.

Association called to order at the hour appointed.

President Cushing presided.

Minutes read and approved.

No business being presented for consideration, the discussion of the second subject was resumed.

The Committee upon Ethics reported the following:

Your committee, to whom was referred the charges preferred at the last annual meeting against Drs. J. Cheesbrough, and J. B. Beauman, for unprofessional conduct, would respectfully report, That in the case of Dr. Cheesbrough, we find him guilty upon his own admission; but in view of the commendable spirit exhibited before the committee, and the fact that he was surrounded at the time by circumstances peculiarly perplexing, and that he pledges himself to a different course in future, we recommend that no further action be had in his case, believing him still worthy of the fraternal regard of his professional brethren.

Dr. J. B. Beauman did not appear before your committee, although duly notified to do so, thus treating this association with contumely, and there being abundant evidence of unprofessional conduct on his part, we recommend that his name be stricken from the roll of membership.

G. W. Keely,

W. H. Morgan,

J. Taft,

} *Committee.*

Which was received and adopted.

Adjourned to meet at 1½ P. M.

AFTERNOON SESSION, 1½ P. M.

Association called to order.

Minutes read and approved.

On motion it was agreed to go into election of Officers, which resulted in the selection of the following:

*President*—Dr. A. M. Moore, Lafayette, Ind.
*Vice-President*—Dr. J. P. Ulrey, Rising Sun, Ind.
*Recording Secretary*—Will Taft, Cincinnati.
*Corresponding Secretary*—N. W. Williams, Xenia, O.
*Treasurer*—J. G. Cameron, Cincinnati.
*Executive Committee*—A. Berry, R. L. Evans, W. H. Morrill.

The President was conducted to the Chair, and made a brief address. Dr. Jno. Allen was now invited to read before the Association a paper on Artificial Dentures. In compliance with the above the Dr. read a very instructive and interesting paper, showing some beautiful specimens of continuous gum work by way of illustration.

On motion, the society tendered a vote of thanks, and requested a copy of his paper for publication.

Dr. McClelland then addressed the Association, giving some practical illustrations of his method of manipulating Rose Pearl. After which a vote of thanks to Dr. McClelland, proposed by Dr. Sedgwick was passed.

Moved to adjourn till 7 o'clock.

EVENING SESSION, 7 P. M.

President Moore in the Chair.

Dr. G. H. Cushing, Secretary *pro tem.*

Operative Dentistry being the topic for discussion, was duly considered and participated in by most of the members present.

The following resolution was offered by Dr. J. A. McClelland:

WHEREAS, It is too much the custom of Dentists to examine teeth, and give advice free of charge, thus not only cheapening our professional services as such, but encouraging "shopping about" of persons who have no idea of employing our services; but having confidence in our judgment, desire our opinion, more to know whether the Dentist of their selection renders them full service or not. And, Whereas, the faithful and conscientious Dentist frequently can not make a thorough examination and give such advice as his cultivated judgment dictates, without the employment of much valuable time.

*Resolved*, That this Association recommend its members to charge a reasonable fee for examinations and advice, where no operation is performed. Carried.

The following resolution was offered by Dr. McCullum:

That the Mississippi Valley Dental Association appropriate the sum of one hundred and fifty dollars to the Ohio

Dental College Association, to be applied to repairing the College building. Carried.

Bill of J. Taft, for issuing notices of meeting. Allowed, and ordered to be paid.

Appropriation to Janitor ordered of fifteen dollars.

The Chair announced the following committee for the ensuing year.

*Dental Ethics*—G. W. Keely, W. H. Morgan, H. A. Smith.
*Appliances*—N. W. Williams, W. H. Shadoan, A. A. Blount.
*Membership*—A. Berry, F. H. Rehwinkel, Sam'l Wardle.

The following delegates to American Dental Association were duly appointed:

Drs. G. W. Keely, Wm. H. Sedgwick, W. F. Morrill, R. A. Mollyneaux, H. McCullum, A. W. Moore, J. P. Ulrey, H. A. Smith, J. A. McClelland, E. C. Sloan.

Adjourned to first Wednesday in March, 1870.

